# Animal and Human Vaccines against West Nile Virus

**DOI:** 10.3390/pathogens9121073

**Published:** 2020-12-21

**Authors:** Juan-Carlos Saiz

**Affiliations:** Department of Biotechnology, Instituto Nacional de Investigación y Tecnología Agraria y Alimentaria (INIA), 28040 Madrid, Spain; jcsaiz@inia.es; Tel.: +34-913471497

**Keywords:** flavivirus, West Nile virus, human vaccines, animal vaccines

## Abstract

West Nile virus (WNV) is a widely distributed enveloped flavivirus transmitted by mosquitoes, which main hosts are birds. The virus sporadically infects equids and humans with serious economic and health consequences, as infected individuals can develop a severe neuroinvasive disease that can even lead to death. Nowadays, no WNV-specific therapy is available and vaccines are only licensed for use in horses but not for humans. While several methodologies for WNV vaccine development have been successfully applied and have contributed to significantly reducing its incidence in horses in the US, none have progressed to phase III clinical trials in humans. This review addresses the status of WNV vaccines for horses, birds, and humans, summarizing and discussing the challenges they face for their clinical advance and their introduction to the market.

## 1. Introduction

Pathogen inoculation as a preventive measure against infectious diseases (namely smallpox) had been used for centuries in China and other parts of the world. However, and although other researchers had applied similar principles before, the credit for the first effective vaccine goes to Edward Jenner who, in 1798, published the first evidence that supported its efficacy in preventing smallpox [1]. Since then, vaccine development and implementation have been continuously growing until became a breakthrough for animal and human health. Nevertheless, and even though vaccination campaigns are saving millions of lives yearly, nowadays, significant challenges still remain in the vaccinology field, as is exemplified by the devastating current SARS-CoV-2 pandemic. For example, the need: (i) for new technologies and adjuvants that allow faster development of more effective, stable, and low-cost vaccines; (ii) that the population is duly informed about the benefits of vaccines as important for their well-being and health, counteracting the increase in the dissemination of opinions of anti-vaccine groups and deniers observed in recent years with data and arguments easily understood by the population to, in this way, obtain their informed consent; (iii) to maintain investment in this field, regardless of the perception of its relevance for the global health of society as a whole and pharmaceutical concerns for the return on investment, thus avoiding situations such as those experienced in the case of Ebola vaccines, for which several candidates have demonstrated their efficacy in animal models, but none has been authorized, which will hamper its control against possible new outbreaks; and (iv) to establish a global vaccine development fund since the current business model only prioritizes those with great market potential [2].

Among the main targets of viral vaccination are (re) emerging pathogens that have caused recent epidemics/pandemics around the world, such as avian influenza, Ebola, SARS, MERS, Chikungunya, Zika, Dengue, and West Nile viruses, as well as those that may pose a threat in the future. This review describes the status of West Nile virus (WNV) vaccines for horses, birds, and humans, summarizing and discussing the challenges they face in their clinical advance and introduction to the market.

## 2. West Nile Virus

WNV is an enveloped flavivirus (family *Flaviviridae*) transmitted by mosquitoes, mainly of the *Culex pipiens* L. complex, whose natural hosts are birds [3]. The virus occasionally infects other vertebrates, mainly equids and humans, which are accidental “dead-end” hosts because viremias achieved in mammals are usually inadequate to maintain the virus cycle, being not enough high to infect a naïve mosquito while feeding on them [4]. Nevertheless, WNV infections in humans and horses have great economic and health repercussions. Although most WNV infections in humans are asymptomatic, around 20% may cause West Nile fever and less than one percent West Nile neuroinvasive disease, which may result in febrile illness, meningitis, encephalitis, flaccid paralysis, and even death, which can occur in around 10% of severe cases [5,6]. In fact, WNV is the arthropod-borne human pathogenic virus with the largest distribution and one of the major causes of human viral encephalitis worldwide [5,6].

WNV is classified into several lineages that do not consistently correlate with its geographical distribution, but only lineages 1 and 2 have been involved in human outbreaks [7]. Early reports suggested that both lineages had differences in pathogenicity, virulence, viremia, the clinical course of infection, and mortality. This initial hypothesis was based mainly on the lack of clinical incidence of WNV in Africa, where only mild diseases have been reported and no deaths have been documented in humans, and where lineage 2 was restricted until it colonized Europe [8]. However, later data in humans and in naturally or experimentally infected animals dismantled this hypothesis. Thus, a study of 644 Greek individuals, which provided a suitable blood sample and lived in an area suffering a WNV lineage 2 epidemic, showed that 5.8% were seropositive for WNV-specific IgG and approximately 18% of them presented clinical manifestations of WNV disease, figures similar to those of patients infected with lineage 1 [9]. Likewise, falcons and magpies experimentally infected with strains of lineage 1 or 2 showed similar mortality rates [10,11]. Even more, studies performed in vaccinated animals showed a high degree of cross-protection between both lineages. Mice immunized with the inactivated Duravaxyn WNV vaccine or with an experimental RSP (Recombinant Subviral Particle) candidate, both based on lineage 1 strains, were protected against exposure to lineage 2 strains [12,13]. Similarly, horses experimentally vaccinated with the ALVAC (^®^)-WNV vaccine [14], or vaccinated under field conditions with the inactivated Equip vaccine [15], both based on lineage 1 strains, were also protected against challenge with heterologous strains of lineage 2.

WNV genome is a single-stranded RNA molecule of positive polarity that encodes three structural (E, prM/M, and C) and seven non-structural (NS1, 2A, 2B, 3, 4A, 4B, and 5) proteins [3]. Among the structural proteins, the E glycoprotein, which is involved in receptor binding, viral entry, and membrane fusion, is the most immunogenic one [16]. This protein has three domains (DI, DII, and DIII), being DIII an immunoglobulin-like structure that contains multiple epitopes recognized by neutralizing antibodies [3]. In fact, several flaviviruses share common epitopes recognized by cross-reactive neutralizing antibodies [17], which may have consequences for the implementation of vaccines, mainly in regions where several of them co-circulate.

## 3. Vaccines

Despite the great efforts invested in recent years in the development of prophylactic measures against this pathogen, there is currently no specific drug or therapy licensed for its treatment [18,19]. However, several candidate vaccines have been successfully developed, some of which have been licensed for use in horses but, despite the fact that no adverse events or safety concerns have been reported in the few clinical trials conducted, none has been authorized for humans. For the development of the WNV vaccine, all possible approaches available have been tested, from purified inactivated and live attenuated viruses, to candidates based on nucleic acids (DNA or RNA), virus-like particles, subunit elements, and recombinant viruses.

### 3.1. Animal Vaccines

As commented before, equids are sporadically infected by WNV and, although in most cases they remain asymptomatic, around 20% can develop clinical signs that use to be more severe than in humans and have important health and economic consequences [20]. When present, signs can range from fever to infection and inflammation of the nervous system, and can cause ataxia, hind and forelimb weakness, quadriplegia, paresis, seizures, chewing and paralysis of the tongue, depression, and ophthalmologic manifestations. [20,21]. Therefore, great efforts have been made to develop and implement equine vaccines. Four of the six licensed vaccines are currently on the market for use in horses. The WN-Innovator, with a classic inactivated whole virion-based approach, was the first to be developed and was licensed by the USDA in 2003 [22]. Live attenuated recombinant viruses have also been used (either based on canary poxvirus or yellow fever virus), as well as a plasmid DNA vaccine, which was the first licensed by the USDA [23], although it was subsequently withdrawn from the market by the manufacturers. All these vaccines are shown to be protective and their use has contributed greatly to reducing the incidence of the disease in horses in the US [23,24]. However, despite their proven efficacy, these vaccines still exhibit some limitations, as the need for repeated administrations to get a solid initial immunization, and the relatively short duration of the induced immunity, which makes necessary annual boosters.

Birds are the natural hosts of WNV and play a key role in the epidemiology of the virus, being many species susceptible to the infection, particularly corvids [4,25]. The disease shows up due to virus invasion of different organs: liver, spleen, kidney, heart, and mainly the central nervous system, and can lead to death within 24–48 h later [26,27]. Several commercial and experimental vaccine candidates have been assayed in wild and domestic birds, although not one has yet been authorized for use on them [27]. Overall, they induced humoral and, although less analyzed, cellular responses, and reduced disease, injury, viremia, viral shedding, and mortality associated with WNV. Furthermore, if they induce herd immunity, they could help prevent outbreaks and the spread of the virus. For example, prospective vaccination of the entire population of California condors (*Gymnogyps californianus*), an endangered species, before the arrival of WNV would have helped prevent infection and its possible extinction [28]. Likewise, vaccination also greatly reduced virus incidence in domestic geese in Israel [29]. However, the implementation of bird vaccines faces several drawbacks, as the feasibility of access to the target host, mainly for wild species, and the administration route. In any case, its availability could benefit domestic populations (farm birds, including those for hunting and restocking activities), as well as wild ones (as those housed in rehabilitation centers and wildlife reserves, and in recreational facilities, like zoos) [27].

### 3.2. Human Vaccines

As mentioned above, human WNV outbreaks can have serious health repercussions that the availability of licensed vaccines would help to minimize.

Human vaccines must be cost-effective, protective, and safe, especially for the most vulnerable populations, like the elderly and the immunosuppressed, whose numbers are increasing around the world. Ideally, vaccines should also be strongly immunogenic and long-lasting with a single dose. Furthermore, although neutralizing antibodies are currently the most reliable protective correlate for flavivirus infections, vaccines should also include determinants that stimulate a balanced T-cell response, essential for providing an effective protective response against infection [30].

Nowadays, effective licensed vaccines, either attenuated (yellow fever, Dengue, and Japanese encephalitis), or inactivated (Japanese encephalitis, tick-borne encephalitis, and Kyasanur forest disease), are available against several flaviviruses. Nevertheless, none has been licensed for human use against WNV, and none of the six vaccines assayed in humans have progressed further than to phase I/II clinical trials [31]. Even more, only two attenuated recombinant candidates expressing the WNV prM and E proteins, ChimeriVax (in a yellow fever virus backbone) and rWN/DEN4Δ30 (in a truncated Dengue virus 4 backbone), induced strong immunity after a single dose [32,33]. Noteworthy, the immunogenicity of these vaccines has been analyzed based on seroconversion and detection of neutralizing antibody, and the development of a T cell-specific response has hardly been addressed.

Therefore, for the implementation of human vaccines, several factors must still have to be analyzed in depth. Among them, the possibility that they induce disease associated with pre-existing immunity to heterologous flaviviruses should be avoided, as the phenomenon of antibody-dependent enhancement (ADE) that can arise from the binding of antibodies that cannot neutralize the virus, and can lead to increased uptake of virus into host cells through Fc receptor-mediated endocytosis in macrophages. Consequently, although its role in the pathogenesis of flavivirus infections other than Dengue remains controversial [34], ADE might lead to more severe symptoms during a secondary, heterologous infection, as it may happen in dengue virus infections [35]. Although it has been reported that DIII does not induce ADE for other flaviviruses [36,37], this hypothetical drawback could be resolved by modifying protein E with mutations in its DIII domain, or nearby, to reduce the binding of antibodies induced against other flaviviruses [38,39]. In any case, addressing and excluding this possibility is necessary during WNV vaccine development.

### 3.3. Current Challenges for Human VACCINES Implementation

Several issues hamper the implementation and introduction of WNV vaccines into the market, such as scientific challenges, safety considerations, difficulties in setting up clinical trials, and cost-effectivity issues, among others.

As mentioned, one of the main scientific questions to solve is the induction of a complete and long-lasting protective immunity that, although seroprevalence studies and experimentation with animal models suggest that this is the case, still needs to be verified in real conditions. In addition, ideally, vaccines should also include determinants that induce a strong cellular response. Likewise, it is desirable that immunization will be induced after the administration of a single dose, and, if possible, that vaccines are marked, that is, that they are DIVA (Differentiating Infected from VAccinated individuals), which could have a considerable impact on blood donation.

Further, clinical trials are difficult to conduct because WNV outbreaks occur sporadically and often unpredictably in regions where other cross-reactive flaviviruses co-circulate. Hence, it becomes difficult to properly assess the impact of candidate vaccines and their implementation strategies.

In addition, another fact to take into account is the possibility of the imposition of new antigenic variants on a previous dominant one, as exemplified by the lineage 1 WN02 strain that has replaced the original lineage 1 NY99 strain in the USA [40]. Although WNV has to infect and replicate in different hosts, making it difficult for mutations to fixate on its genome because they can be beneficial for infecting one host (mosquitoes) but detrimental to others (birds), vaccine production must be conscious of the possibility that new antigenic variants appear and are imposed in order to minimize their possible consequences.

Ultimately, vaccines should be inexpensive, but keep in mind that, while still debated, the few studies conducted so far have concluded that current WNV vaccines are unlikely to save costs [41,42].

## 4. Conclusions

WNV remains a significant threat to humans, equids, and birds in many parts of the world, as demonstrated by the worrisome increase of cases reported in the E.U. in recent years. An accumulated number of 285 human cases and 31 deaths were reported to the European Centre for Disease Prevention and Control (ECDC) in 2020 [43]. This increase is favored, among other reasons, by the introduction or imposition of new lineages, the increase in temperature that can favor the colonization of new regions by mosquito vectors competent for their transmission, and the globalization of trade and the transport of people and animals. All this makes it necessary to have effective and inexpensive vaccines for immediate use.

As has been detailed, the technologies to produce vaccines are already available and have proved to be safe and protective in the few clinical trials conducted. In this regard, and even though clinical trials are difficult to establish, vaccination in restricted regions where outbreaks appear could help not only to combat the spread of infection but also to get a better idea of the performance of vaccines in terms of protection, the durability of immunity, etc. In any case, some issues should be improved and properly addressed to facilitate its introduction into the market. Among them are a solid demonstration of the induction of a long-lasting immunity, preferably after a single immunization, the production and validation of marked candidates, the exclusion of the possibility of inducting a putative ADE phenomenon, its safety in elderly and immunocompromised people, and the optimization of their production to reduce their costs. To solve these issues, a joint effort of the different agents involved (pharmaceutical companies, governments, international institutions, and non-governmental organizations) to establish a common fund for its development, clinical trials implementation, and the establishment of adequate vaccination strategies would be desirable.
